# Associations between clinical data and computed tomography features in patients with epidermal growth factor receptor mutations in lung adenocarcinoma

**DOI:** 10.1007/s10147-017-1197-8

**Published:** 2017-10-07

**Authors:** Yiyuan Cao, Haibo Xu, Meiyan Liao, Yanjuan Qu, Liying Xu, Dongyong Zhu, Bicheng Wang, Sufang Tian

**Affiliations:** 1 Radiology Department, Zhongnan Hospital of Wuhan University, Wuhan University, Wuhan, China; 2 Pathology Department, Zhongnan Hospital of Wuhan University, Wuhan University, Wuhan, China

**Keywords:** Computed tomography, Epidermal growth factor receptor, Shadow disappearance rate, GGO, Emphysema

## Abstract

**Background:**

To analyse the differences in computed tomography (CT) features between patients with lung adenocarcinoma who have epidermal growth factor receptor (EGFR) mutations and those who have wild-type EGFR.

**Methods:**

Patients with lung adenocarcinoma (*n* = 156) were enrolled from October 2013 to March 2016, including 56 patients with wild-type EGFR and 100 patients with EGFR mutations. Two independent radiologists evaluated patient characteristics and imaging features. Chi-squared test, Fisher’s exact test or ANOVA was applied to discriminate clinical and CT characteristics between the genotypes. A prediction tool for EGFR mutation was devised from principal component analysis.

**Results:**

The proportion of females and non-smokers in the exon 19 deletion and exon 21 missense groups was higher than in the wild-type group (*P* < 0.01). Severe emphysema was higher in the wild-type group than in the exon 19 deletion group (*P* < 0.01). The maximum diameter in the mediastinal window (MaxD_mediastinal_) in the wild-type group was longer than in the exon 19 deletion and exon 21 missense groups. The minimum diameter in the mediastinal window (MinD_mediastinal_) in the wild-type group was also longer than in the exon 21 missense group, with a significant difference (*P* < 0.05). The tumor shadow disappearance rate (TDR) in the exon 19 deletion group was higher than in the wild-type group. Ground glass opacity (GGO) appeared to be more common in the exon 19 deletion group (*P* = 0.010). The prediction score for exon 19 deletion mutation was: 0.305 × gender + 0.254 × smoking history + 0.198 × MaxD_mediastinal_ + TDR × 0.254 + 0.280 × GGO + 0.095 × emphysema. The sensitivity and specificity for predicting exon 19 deletion were 59.09 and 76.79%, respectively. The prediction score for the exon 21 missense mutation was: 0.354 × gender + 0.291 × smoking history + 0.410 × MaxD_mediastinal_ + 0.408 × MinD_mediastinal_. The sensitivity and specificity for predicting exon 21 missense mutation were 72.34 and 78.57%, respectively.

**Conclusion:**

As well as gender, smoking history and GGO, adenocarcinomas with EGFR mutation were significantly associated with emphysema, TDR, and the diameter in the mediastinal window. As exon 19 deletion and 21 missense mutations might be predicted by those features, the scoring system might be valuable for clinical diagnosis.

## Introduction

Lung and bronchus cancer is the leading cause of cancer-related deaths worldwide, with an approximate 0.16 million deaths in the United States annually [1], and 0.61 million deaths per year in China [2]. Lung cancers are classified by histologic features as non-small cell lung cancer (NSCLC) and small cell lung cancer (SCLC). NSCLC accounts for 85−90% of lung cancers. Most of these patients present with inoperable advanced or metastatic disease with an extremely poor prognosis. The development of molecular-targeted therapies has revolutionized NSCLC therapy by affording better tumor control and selectivity with less toxicity than traditional chemotherapy [3].

Epidermal growth factor receptor (EGFR) is a transmembrane receptor tyrosine kinase involved in the signaling pathways that regulate cell proliferation, apoptosis, angiogenesis, and invasion [4–6]. Ninety-five percent of EGFR mutations are found in adenocarcinomas, which are the most common histologic type of NSCLC [7]. EGFR mutations with exon 19 deletions and L858R point mutation in exon 21 occur most frequently in NSCLC, and have a high response rate of approximately 70% to EGFR tyrosine kinase inhibitor (TKI) therapy [4, 6, 8, 9]. Exon 20 insertions are also well described and have been associated with TKI resistance [10, 11], representing 4–9% of EGFR-mutant lung cancers [11–13]. Exon 20 point mutations like T790M are rarely identified in pretreatment NSCLC tumors [11, 13, 14], but can be found in >50% of NSCLC tumors which have acquired resistance to TKI therapy [11, 14]. Some uncommon point mutations such as exon 21 (at L861) and exon 18 (at G719) were found to have a response rate to EGFR-TKI therapy of approximately 50% [11, 13]. However, wild-type NSCLC has been reported to be less sensitive to TKI therapy [15].

It would be very useful with regard to treatment if the EGFR mutation status of NSCLC could be identified without molecular examination. To our knowledge, only a few studies have attempted to determine the relationship between imaging features and molecular findings. Computed tomography (CT) imaging plays an important role in diagnosis and response assessment in cases of NSCLC. Therefore, the purpose of this study was to retrospectively identify CT features that correlate with EGFR mutation status in lung adenocarcinomas in a cohort of East Asian patients.

## Materials and methods

### Patient selection

This retrospective study was approved by the institutional review board. Informed consent was waived. From October 2013 to March 2016, a search of the electronic medical records at Zhongnan Hospital of Wuhan University revealed that 385 patients had undergone EGFR mutation testing.

Exclusion criteria were (1) no preoperative CT on the Picture Archiving and Communication System (PACS, Neusoft Vision 3.1) including both non-contrast-enhanced CT and contrast-enhanced CT, (2) non-adenocarcinoma cell type, (3) cases with multiple lesions on CT which individually could not be conclusively correlated with the lesions documented in the pathology report, (4) difficult to contour the tumor margin on CT images, (5) incomplete electronic medical records, and (6) the specimens were not obtained from the lung resection or open lung biopsy. Therefore, 229 patients were excluded.

Clinical and pathological data collected for analysis included age, gender, stage, smoking status (nonsmoking defined as patients who had never smoked), and emphysema status (the severity of emphysema was graded according to Goddard’s Grade [16] and categorized into two groups). Tumor staging was according to the American Joint Committee on Cancer Staging Manual, 7th edition [17].

### Histologic evaluation and molecular analysis

All histological specimens were formalin-fixed and stained with hematoxylin–eosin as part of the routine regulations of our hospital. Two board-certified pathologists (BW with 18 years experience of pathologic diagnosis of lung cancer, and ST with 17 years experience of pathologic diagnosis of lung cancer) reviewed the pathologic specimens and recorded the pathologic type of each tumor according to the 2015 WHO Classification of Lung Tumors [18].

All cases were analyzed for EGFR mutations at exons 18–21 using a pyrosequencing assay based on polymerase chain reaction (PCR). The sequence analysis was performed using a real-time quantitative PCR system (Mx3000P; Stratagene, La Jolla, CA, USA).

All cases underwent EGFR mutation analysis. The specimens were formalin fixed and paraffin embedded. Genomic DNA was extracted from the tumor tissue and EGFR mutations in exons 18 (G719X), 19 (deletion), 20 (T790M, 20-Ins and S768I), and 21 (L858 and L861Q) were detected using a fluorescence PCR diagnostic kit (Amoy Diagnostics Co., Xiamen, China). All EGFR mutation analysis was performed in the pathological laboratory by a board-certified pathologist (ZX with 10 years experience of pathologic diagnosis of lung cancer). The intensity score was defined as—cycle threshold (Ct) scores 0–26 were considered strong positive expression, 26–29 were considered weak positive expression, and >29 were considered negative expression.

### CT imaging

All patients included in the study had preoperative chest CT scans available, including both pre-contrast-enhanced and contrast-enhanced scans, which had been conducted within 1 month prior to surgery. CT imaging was performed using one of the 2 CT systems (Sensation 16 and Somatom Definition; Siemens Medical Systems, Erlangen, Germany). For the Sensation 16, the parameters were 120 kV and 100 mA, with dose modulation. Images were reconstructed with a section thickness and an interval of 5 mm without a gap using the B50 algorithm and with a section thickness of 1 mm using the B80 algorithm. For the Somatom Definition, the parameters were 120 kV and 100–400 mA, with dose modulation. Images were reconstructed the same as Sensation 16. Contrast-enhanced images were obtained after intravenous administration of iopromide with 300 mg of iodine/mL (Ultravist 300; Bayer Pharma, Berlin, Germany) at a rate of 3.0 mL/s using a high-pressure syringe, with a dose based on patient weight (70–120 mL). CT scanning was performed with a 45-s delay.

### CT interpretation

All images were viewed at mediastinal (width 350 HU, level 50 HU) and lung window (width 1500 HU, level −700 HU) settings for axial images on PACS. Two board-certified thoracic radiologists interpreted the CT images. Both radiologists (YC and YQ had 9 and 19 years of experience, respectively, in chest image interpretations) were aware that the patients had lung cancer, but were blinded to the pathologic diagnosis as well as the EGFR status.

The imaging characteristics of the primary lesions were recorded. In terms of morphologic characteristics, we calculated the tumor size, TDR, and the relative enhancement (Erel). The presence or absence of calcification, air bronchograms, bubble-like lucency or cavities, vessel convergence sign, lobulation, pleural retraction, GGO, pneumonia-like consolidation, spiculation, pleural effusion and pericardial effusion was assessed. TDR = 1-MaxD _mediastinal_ × MinD _mediastinal_/(MaxD _lung_ × MinD_Lung_), where MaxD _mediastinal_ and MinD _mediastinal_ meant the length of the longest and shortest diameter in millimeters in the mediastinal window, and the MaxD _lung_ and MinD_Lung_ meant the diameter in the lung window [19]. Erel = (Apost—Apre)/Eart, where Apost was contrast-enhanced CT attenuation of the lesion, Apre was unenhanced CT attenuation of the lesion and Eart was the attenuation of the aescending aorta in contrast-enhanced CT; these (regions of interest) ROIs were all in the same transverse section [20]. Lobulation was defined when a portion of the surface of a lesion showed a shallow wavy configuration (except for regions abutting the pleura) and was calculated by numbers. Pneumonia-like consolidation is described as a homogenous opacity lesion in the lung defined by little or no volume loss, effacement of blood vessel shadows, and sometimes by the presence of an air bronchogram, and no concomitant bacterial pneumonia or obstructive pneumonia due to an exophytic lesion occluding the lumen of the main or lobar bronchi [21].

All the counting and measurement values took the average of the two radiologists. The differences were between two readers in the imaging interpretation of the presence of air bronchogram (1/156, 0.6%), vessel convergence sign (3/156, 1.9%), and pneumonia-like consolidation (1/156, 0.6%). Final conclusions regarding the CT examination findings were reached in consensus.

### Statistical analysis

All statistical analyses were performed using SPSS (version 21.0; SPSS Inc., IBM Co., Chicago, IL, USA). Categorical variables or continuous variables were analyzed by chi-squared test, Fisher’s exact test, ANOVA or Welch’s ANOVA, as appropriate. All reported *P* values were two-sided, and a *P* value <0.05 was considered as statistically significant. In intergroup chi-squared tests, the Bonferroni correction was used to counteract the problem of multiple comparisons; the *p* value was 0.01.

When the mono-factor analysis was complete, multivariable regression analysis was performed, after analysis of the multi-collinearity. A prediction tool for EGFR mutation was devised from the principal component analysis, which took the data to draw receiver operating characteristic (ROC) curves and calculate the available area under the curve (AUC). The Youden index was used to calculate the optimum cut-off.

## Results

### Patient characteristics

A total of 156 patients with adenocarcinomas were included in the analysis. One hundred patients harbored an EGFR mutation—exon 21 missense in 47 patients (47.0%), exon 19 deletion in 44 (44.0%), exon 20 insertion in 6 (6.0%) and exon 18 deletion or missense mutation in 3 patients (3.0%). One hundred and twenty-one patients had lung resection and 35 had open lung biopsy. EGFR mutation was seen more frequently in female patients (57/100, *P* < 0.001), non-smokers (78/100, *P* < 0.001), and normal and less severe emphysema patients (95/100, *P* = 0.016). There were no differences in age or tumor stage between wild-type and subtype of EGFR-mutant lung adenocarcinomas (Table 1). After comparing the wild-type and each subtype of the mutation groups, the proportion of females and non-smokers in the exon 19 deletion and exon 21 missense groups was higher than in the wild-type group (*P* < 0.01). Severe emphysema was higher in the wild-type group than in the exon 19 deletion group (*P* < 0.01) (Table 2).

**Table 1:** Clinical characteristics of wild-type and mutation subtypes

Characteristics	Total	Wild-type	Mutation				*P*
			18	19	20	21	
Age (years)		61.482 (41–80)	56.000 (52–62)	60.136 (32–82)	58.167 (46–76)	63.128 (38–77)	0.309
Gender							0.000
Male	90	47	2	17	3	21	
Female	66	9	1	27	3	26	
Smoking history							0.000
Smoker	65	43	2	8	3	9	
Non-smoker	91	13	1	36	3	38	
Stage							0.639
IA	37	11	1	11	2	12	
IB	31	8	1	7	0	15	
IIA	8	5	0	2	1	0	
IIB	15	8	0	4	0	3	
IIIA	33	14	1	9	2	7	
IIIB	4	2	0	2	0	0	
IV	28	8	0	9	1	10	
Emphysema							0.016
Normal and score <8	140	45	2	43	5	45	
Score ≥8	16	11	1	1	1	2

**Table 2:** Comprehensive tables with statistically significant differences

Characteristics	Wild-type	Mutation				*P*
		18	19	20	21	
Gender						0.000
Smoking history						0.000
Emphysema						0.008
MaxD mediastinal						< 0.05
TDR						< 0.05
GGO						< 0.05
Gender						0.000
Smoking history						0.000
MaxD mediastinal						< 0.05
MinD mediastinal						0.000
Erel						< 0.05
Erel						< 0.05

### CT imaging evaluation

The Erel in the exon 18 deletion or missense mutation groups was lower than in the 19 deletion and wild-type groups (*P* < 0.05) (Tables 2, 3). The initial tumor size was measured in both the mediastinal window and the lung window. There were statistical differences between the wild-type group and EGFR mutation group in MaxD_mediastinal_ and MinD_mediastinal_. The MaxD_mediastinal_ in the wild-type group was longer than in the exon 19 deletion and exon 21 missense groups, and the MinD_mediastinal_ in the wild-type group was longer than in the exon 21 missense group, with a significant difference (*P* < 0.05) (Tables 2, 3). The TDR in the exon 19 deletion group was higher than in the wild-type group. GGO appeared to be more common in exon 19 deletion patients (*P* = 0.010). According to the statistics, there were no significant differences between the groups with regard to the location of the lesions, attenuation, pneumonia-type, shape, boundary, lobulation, presence of spiculation, calcification, cavitation, air bronchogram, vascular convergence sign, pleural indentation, pericardial effusion, and pleural effusion (Table 3).

**Table 3:** The CT features of wild-type and mutation subtypes

Characteristics	Total	Wild-type	Mutation				*P*
			18	19	20	21	
Density (HU)							
Non-enhanced		34.089 ± 9.449	34.333 ± 6.351	33.136 ± 11.235	37.667 ± 8.824	31.936 ± 10.182	0.675
Enhanced		60.946 ± 13.770	51.000 ± 8.660	60.250 ± 14.379	58.833 ± 9.621	57.553 ± 14.359	0.599
Erel		0.180 ± 0.079	0.126 ± 0.013	0.197 ± 0.099	0.166 ± 0.086	0.160 ± 0.065	0.002
Size (mm)							
MaxD mediastinal		39.125 (6–109)	23.000 (11–30)	29.727 (6–75)	26.000 (14–45)	29.894 (3–88)	0.032
MinD mediastinal		31.518 (5–93)	21.000 (11−26)	23.864 (4–52)	22.333 (10–42)	22.809 (2–50)	0.160
MaxD lung		40.714 (7–139)	24.333 (15–30)	34.409 (13–92)	31.167 (15–45)	33.574 (11–101)	0.271
MinD lung		32.875 (6–99)	22.333 (15–26)	27.273 (11–61)	28.000 (12–46)	26.250 (9–52)	0.311
TDR		0.071 (0–0.443)	0.154 (0–0.462)	0.206 (0–0.926)	0.267 (0–0.701)	0.197 (0–0.962)	0.042
Lobulation		4.589 ± 3.431	2.333 ± 2.082	4.341 ± 2.745	6.167 ± 1.835	4.745 ± 3.473	0.509
Homogeneity after contrast							0.125
Heterogeneous	65	30	0	16	3	16	
Homogenous	91	26	3	28	3	31	
GGO							0.034
Present	36	5	1	15	2	13	
Absent	120	51	2	29	4	34	
Pericardial effusion							0.897
Present	5	1	0	2	0	2	
Absent	151	55	3	42	6	45	
Location							0.164
Inferior lobe of left lung	34	17	0	8	0	9	
Superior lobe of left lung	29	8	1	9	2	9	
Middle lobe of right lung	10	2	0	0	1	7	
Inferior lobe of right lung	52	21	1	13	2	15	
Superior lobe of right lung	31	8	1	14	1	7	
Pneumonia-type							0.415
Pneumonia-type	5	0	0	2	0	3	
Non-pneumonia-type	151	56	3	42	6	44	
Shape							0.400
Round or oval	110	40	3	34	4	29	
Irregular	46	16	0	10	2	18	
Spiculation							0.899
Present	108	37	2	30	5	34	
Absent	48	19	1	14	1	13	
Boundary							0.103
Clear	146	52	3	38	6	47	
Obscure	10	4	0	6	0	0	
Calcification							0.435
Present	9	5	0	2	1	1	
Absent	147	51	3	42	5	46	
Cavitation							0.960
Present	27	9	1	8	1	8	
Absent	129	47	2	36	5	39	
Air bronchogram							0.206
Present	38	10	0	7	3	18	
Absent	118	46	3	37	3	29	
Vascular convergence sign							0.116
Present	57	14	2	18	4	19	
Absent	99	42	1	26	2	28	
Pleural indentation							0.652
Present	95	33	1	25	4	32	
Absent	61	23	2	19	2	15	
Pleural effusion							0.381
Present	15	3	0	7	0	5	
Absent	141	53	3	37	6	42	
Mediastinal lymph node metastasis							0.550
Present	57	24	1	16	3	13	
Absent	99	32	2	28	3	34	

### Prediction model of EGFR mutation from CT findings

**Fig. 1:**
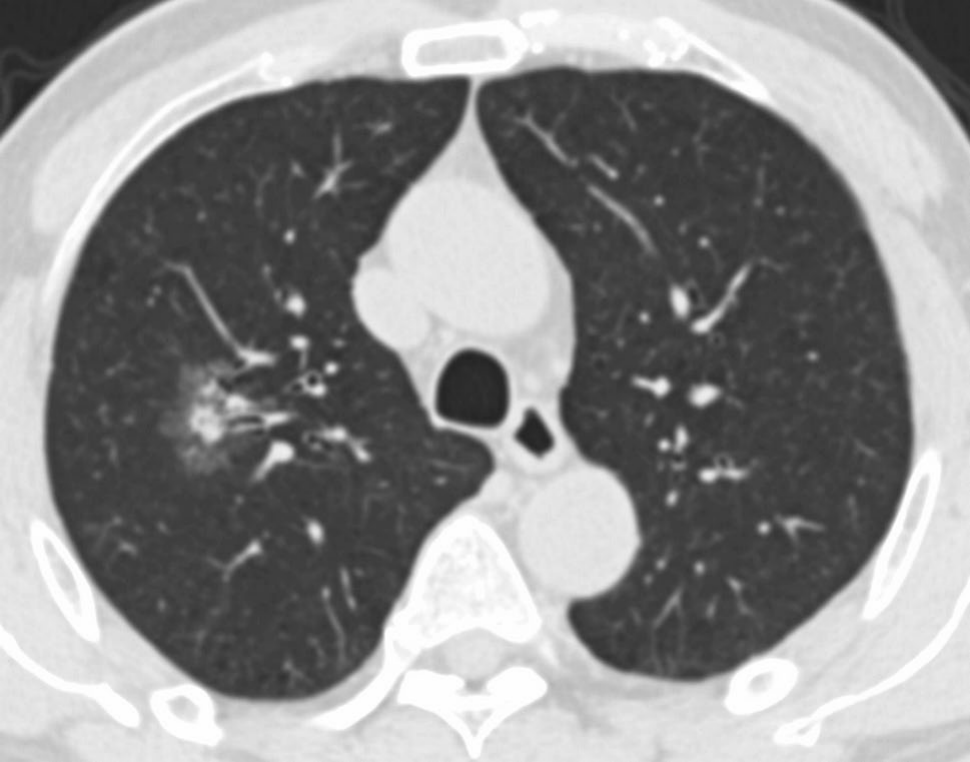
F, 42Y, exon 19 deletion positive, the lesion located in the superior right lobe was a mixed GGO

**Fig. 2:**
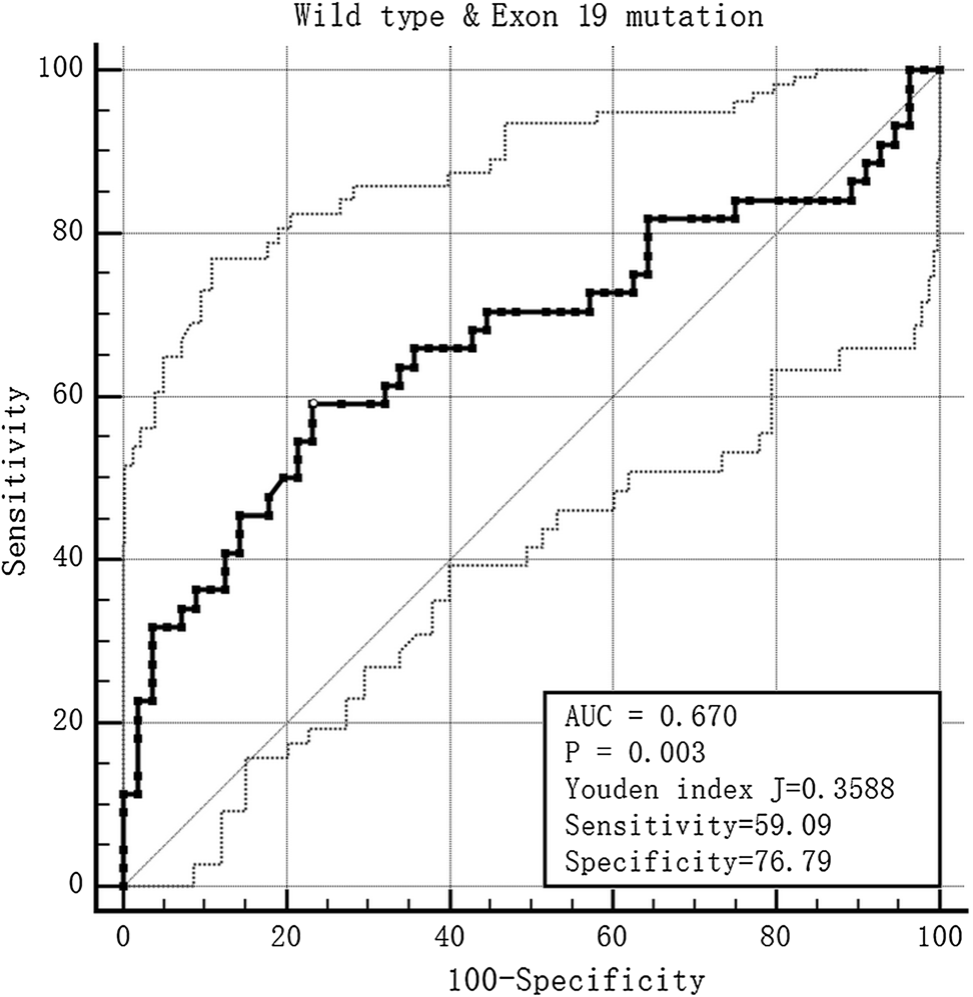
The ROC of the prediction score in the wild-type and exon 19 mutation groups

After the mono-factor analysis, we found gender, smoking history, emphysema, MaxD_mediastinal_, TDR and GGO showed statistical differences between the wild-type group and the exon 19 deletion group (Fig. 1). We performed a collinearity diagnosis of the 6 factors (the condition index was 12.970). We then extracted the main factors by principal component analysis, and the prediction score was calculated by the product sum of the eigenvector and standardized CT and clinical feature variables: 0.305 × gender + 0.254 × smoke history + 0.198 × MaxD_mediastinal_ + TDR × 0.254 + 0.280 × GGO + 0.095 × emphysema. The AUC was 0.670, the Youden index was 0.359, the cut-off value was −0.057, and the sensitivity and specificity for predicting exon 19 deletion were 59.09 and 76.79%, respectively (Fig. 2).

**Fig. 3:**
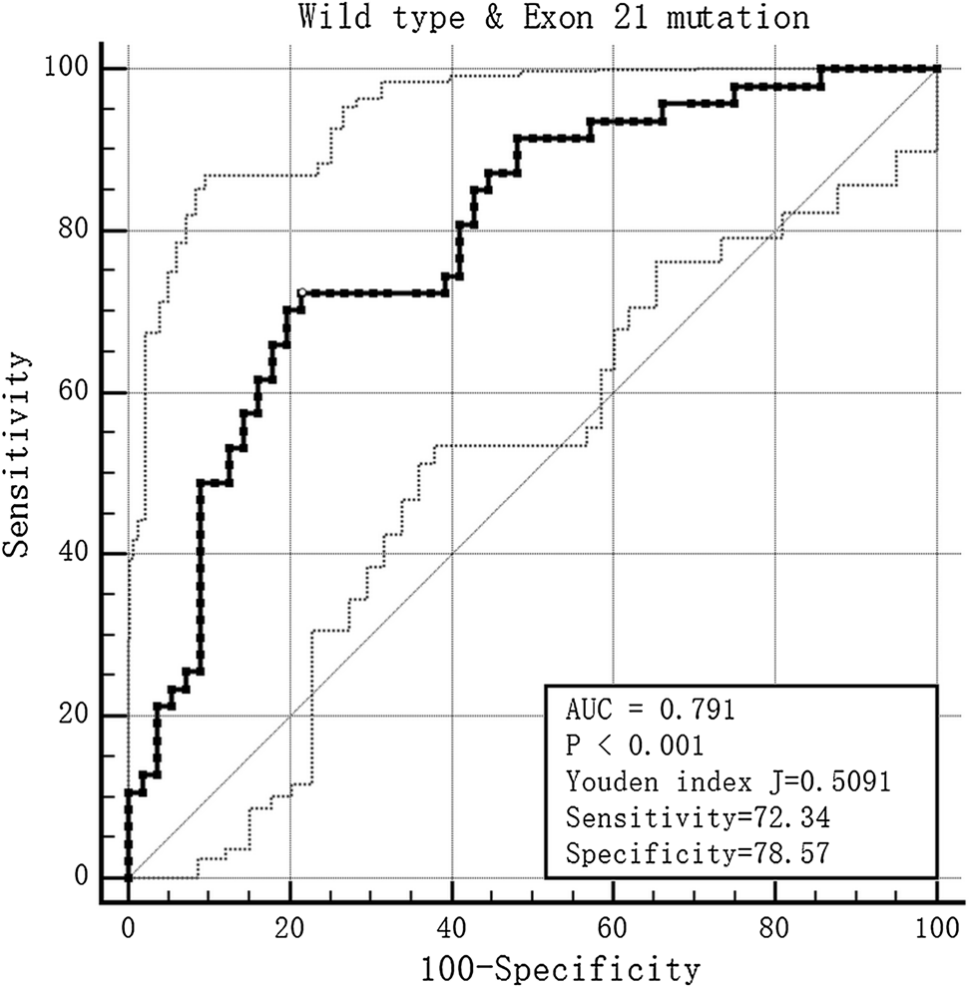
The ROC of the prediction score in the wild-type and exon 21 mutation groups

The condition index of collinearity diagnosis in the wild-type and exon 21 missense groups was 18.074, meaning that there was multiple co-linear relationships between the parameters. We also used principal component analysis to solve the problem. The prediction score was calculated by the product sum of the eigenvector and standardized CT and clinical feature variables: 0.354 × gender + 0.291 × smoke history + 0.410 × MaxD_mediastinal_ + 0.408 × MinD^mediastinal^. The AUC was 0.791, the Youden index was 0.509, the cut-off value was −0.231, and the sensitivity and specificity for predicting exon 21 missense were 72.34 and 78.57%, respectively (Fig. 3).

After regression analysis with wild-type and exon 18 deletion or missense groups in Erel, the *P* value was 0.238, and in the wild-type and exon 18 deletion or missense groups it was 0.242.

## Discussion

There were two main parts to our study. In the first part, we evaluated the differences in various clinical features and CT features between different genotypes in lung adenocarcinoma. The results showed that exon 19 deletion EGFR mutation was associated with gender, smoking history, the MaxD_mediastinal_, TDR, GGO and emphysema grade, and the exon 21 missense mutation was associated with gender, smoking history, and the MaxD_mediastinal_ and MinD_mediastinal_. There were statistical differences between the wild-type and exon 18 deletion or missense groups in Erel, and also between the exon 18 deletion or missense and exon 19 deletion groups. In the second part, we explored the predictive ability of these factors by multiple logistic regression analysis and principal component analysis. The sensitivity and specificity of the prediction score that we devised for exon 19 deletion EGFR mutation were 59.09 and 76.79%, respectively, and for exon 21 missense mutation were 72.34 and 78.57%, respectively. However, the Erel had no prediction value for the diagnosis.

The purposes of the scoring system are as follows. First, the EGFR tyrosine kinase inhibitors have a diverse curative effect in different subtypes of the EGFR mutation, so the scoring system may be useful to predict the curative effect in a non-invasive way. Second, it can be used in those patients who are not suitable for surgery or other invasive operations; this scoring system may be useful in helping to decide the EGFR mutation status. Third, it may avoid unnecessary false-negative results, and help to decide whether the repeat biopsy or surgery is worth performing for patients with inconsistent genetic testing and clinical features.

Our study focused on a population of East Asian origin. Compared to a Western population, a population of East Asian origin with NSCLC had higher rates of EGFR mutations [22, 23]. Our study demonstrated that EGFR mutation lesions were significantly higher in females and in non-smokers. Indeed, we identified EGFR mutations in 64.10% of all cases; female patients comprised 57% in the mutation group, which was consistent with previous studies [20, 24–26], especially in the subtypes of the exon 19 deletion and exon 21 missense groups.

Besides the demographic characteristics, our study had an interesting feature in that there was a relationship between the Goddard scoring system and exon 19 deletion. According to the modified Goddard scoring system [16], it was considered to be a representative value of the severity of emphysema in each person, and was very convenient to apply. In our study, a minimum score of 0 to a maximum score of 8 was considered a representative value of the mild type of emphysema in the lungs; when the score was >8, it was considered as severe emphysema. The results showed the incidence of exon 19 deletion was higher in the mild type (97.73%) than in the wild-type (80.35%). Furthermore, the reason for this difference may be correlated with the cause of the emphysema, as smoking is a major risk factor for the development and progression of chronic obstructive pulmonary disease and lung cancer [16, 27, 28], and the EGFR mutation lesions were significantly higher in females and in non-smokers. We propose that the present results may be valuable in clinical practice, because to define emphysema using the Goddard scoring system is very easy to apply, requires little clinical testing, and can provide complementary information for diagnosing.

Most of the CT features had no significant statistical differences. We demonstrated that MaxD_mediastinal_, TDR, and the presence of GGO showed statistical differences between the exon 19 deletion and wild-type groups, and MaxD_mediastinal_ and MinD_mediastina_ showed statistical differences between the exon 21 missense and wild-type groups. The relationship between GGO and EGFR has been frequently studied [6, 20, 24–26]. GGO appeared to be more common in EGFR mutation patients than in wild-type patients. A few articles have reported that the presence of GGO was more significant in exon 21 missense mutation, and no difference was seen in exon 19 deletion and wild-type patients [26]; however, some have reported differences in wild-type and both exon mutations [25]. These conclusions were somewhat out of line with our observations, although it also indicates the diagnostic value of GGO. These differences might be due to the study population and the diagnostic procedures.

TDR has been proposed as a new radiologic variable. It is calculated from the tumor shadow on both the lung and mediastinal windows on CT images, to represent the proportion of the GGO area in the entire lesion [29]. Previous research used semi-automated nodule assessment software and showed that there was no significant difference regarding TDR between the EGFR mutation type and the wild-type [30]. One reason for the differences might be the fact that our study focused on the differences between the wild-type and the EGFR mutation subtype, as well as using a different assessment method. However, to our knowledge, this feature has not been previously reported. It can be used to quantify the shape of the tumor but further studies are needed to test and verify our results.

We measured both the maximum and minimum diameter of the mediastinal window and the lung window. The results showed the MaxD_mediastinal_ of the wild type was longer than the exon 19-deletion group, and in the wild-type and exon 21 missense groups, both the MaxD_mediastinal_ and MinD_mediastinal_ of the wild-type were longer. Previous research also focused on this issue; some studies measured the average diameter of the lesion, and some only measured the diameter in the lung window. Lee et al. demonstrated that in the early stage of lung adenocarcinoma, the diameter of the EGFR mutation tumor was longer than the wild-type; however, the measurement methods were not specified [31]. Usuda et al. measured the maximum tumor diameter in chest CT and the result showed that the diameter in the wild-type group was longer than in the mutation group [32]. Liu et al. [20] measured both the maximum and minimum tumor diameter and also reached the same result as the study by Usuda et al. [20], which was partly in agreement with our result. According to the literature, the criteria for diameter measurement vary widely. Whether or not they are associated with EGFR mutations is also controversial; they might be related to different races and different stages of lung adenocarcinoma. This deserves further study in the follow-up work.

Erel is a relatively standardized enhancement index. Only a few articles focus on this parameter and have shown no statistical significance [20]. Our study showed there were some statistical differences between the wild-type and exon 18 mutation groups, as well as between the exon 18 and 19 mutation groups; however, after logistic regression, there was no statistical difference between the groups. This suggests that Erel has little predictive value in the diagnosis of EGFR mutations. There might be two reasons for this. One reason is that EGFR mutations may have nothing to do with Erel, and the other is that the results might be due to the small sample size; the exon 18 mutation group had only 3 patients which will have had a bearing on the credibility of the results. We may need to collect more relevant cases in the follow-up work to obtain more accurate results.

In our study, we quantitatively measured some of the CT parameters, such as diameter, TDR, Erel, emphysema, and found that gender, smoking history, emphysema, diameter and TDR had some predictive value in wild-type and different EGFR mutation subtypes. We then established a prediction model to help predict whether there is a mutation. Screening some parameters such as the value of Erel has laid the foundation for further research. We also found that Erel had some statistical significance, but because of the small amount of data, further research is needed.

As a retrospective cohort study, our study had several limitations. We used two different CT scanners, so the scanning parameters were not same and this might cause bias to the results. As this study was performed in a single large medical center, there may have been bias in the selection of patients; the sample size was not relatively large and not predetermined in dimension with a power analysis.

To our knowledge, although many researchers have focused on the relationship between the genotype, this study is the first to present and discuss some new findings among EGFR mutation subtype and wild-type lung adenocarcinomas. As well as gender, smoking history and GGO, adenocarcinomas with EGFR mutations were significantly associated with emphysema, TDR, and the diameter in mediastinal window. As some EGFR mutation subtypes might be predicted by these features, the scoring system might be valuable for clinical diagnosis.
